# Editorial: Special Issue, “Molecular Advances in Skin Diseases 2.0”

**DOI:** 10.3390/ijms25115928

**Published:** 2024-05-29

**Authors:** Naoko Kanda

**Affiliations:** Department of Dermatology, Nippon Medical School Chiba Hokusoh Hospital, Kamagari 1715, Inzai 270-1694, Chiba, Japan; n-kanda@nms.ac.jp; Tel.: +81-476-99-1111; Fax: +81-476-99-1909

Recently, the pathomechanisms of various skin diseases have been progressively elucidated. The therapeutic targets of certain skin diseases, whether inflammatory, infectious, or neoplastic, have been rapidly identified, and, thus, novel target-oriented therapies have been continuously developed.

In this Special Issue of *IJMS*, we have published five research articles and six reviews on recent advances in the research of all fields of skin diseases from molecular viewpoints.

Basal cell carcinoma (BCC) is the most common cutaneous malignancy. Recently, solid advances have emerged in unveiling the molecular mechanisms of BCC. In nevoid BCC syndrome, also named Gorlin syndrome, the mutation of the patched 1 gene (*PTCH1*) was identified. *PTCH1* plays a role in the hedgehog pathway, and dysregulations in this pathway are crucial for the pathogenesis of BCC. Hoashi et al. summarized the clinical and pathological features of BCC and discussed therapeutic strategies targeting the hedgehog pathway, p53, or melanocortin-1 receptor and biomarkers classifying BCC subtypes or predicting prognosis, such as nicotinamide N-methyl-transferase or enhancer of zeste homolog 2 [1]. 

COVID-19 is a recently emerged viral infection caused by severe acute respiratory syndrome coronavirus 2 (SARS-CoV-2). Komine et al. reviewed the basic nature of SARS-CoV-2, including the possible emergence pathways, mechanisms of infection, and exacerbation in patients with obesity, hypertension, diabetes mellitus, and pulmonary diseases [2]. The major inflammatory skin diseases, psoriasis and atopic dermatitis (AD), do not seem to cause poor prognosis for COVID-19 based on the epidemiological data so far. It is speculated that in cutaneous inflammatory diseases, even if they have systemic inflammation, the main site of inflammation is located on the skin, which may have a different impact on COVID-19 infection derived from organ specificity. Further investigation is needed to elucidate this difference.

Yamanaka et al. reported the interplay between type 1, type 2, and type 3 lymphocytes and cytokines in AD [3]. In an AD mouse model where *caspase-1* is specifically amplified under *keratin-14* induction, type 1 cells are dominant at all times, whereas type 2 and type 3 cytokine-producing cells are equally present. IL-25-producing cells, overlapping with IL-17F-producing cells, are persistently increased, and may contribute to the prolongation of type 2 inflammation. IL-25 may be a treatment target for AD.

Unsuccessful wound closure is associated with sustained TGF-β production, which makes keratinocytes chronified/senescent type and suppresses their migration. Liarte et al. reported that the amniotic membrane (AM) treatment of chronified keratinocytes re-enabled migration in the early stages of wound healing, also promoting proliferation at later stages. AM treatment promotes the expression of keratinocyte activation marker cytokeratine 17 and proliferation marker Ki67 in HaCaT cells. AM treatment might promote the restoration of proper keratinocyte responses in wound healing [4].

Inverse psoriasis (IP) affects the groin, armpits, navel, intergluteal fissure, and external genitalia. Pietrangelo et al. investigated the efficacy of topical LimpiAL 2.5% for IP. The main component of LimpiAL 2.5% is the patented HAc-40 ingredient made by high-molecular-weight hyaluronic acid conjugated with a bacterial wall fragment from *Cutibacterium acnes*. The LimpiAL treatment gave a beneficial therapeutic effect; it decreased psoriasis area and severity index, dermatology life quality index, and pain vidual analog scale score. LimpiAL increased the biological diversity of the skin microbiota and decreased some *Corynebacterium* species, and increased some *Staphylococcus* species. LimpiAL might improve IP by restoring the eubiosis conditions and modulating the bacterial composition of the resident microbiota [5].

Immune checkpoint inhibitors (ICIs) have been used for the treatment of malignant melanoma. The mechanistic exploration of tumor immune responses is essential to improve the therapeutic efficacy of ICIs. Nakamura and Okuyama reviewed the relations between changes in immune cell repertoire and responsiveness to treatment with ICIs. Since tumor immune responses are based on antigen-specific immune responses, investigators have focused on T cell receptors (TCRs) and have analyzed changes in the TCR repertoire [6]. The proliferation of T cell clones against tumor antigens is detected in patients who respond to treatment with ICIs. The proliferation of these T cell clones is observed within tumors as well as in the peripheral blood. Clonal proliferation has been detected not only in CD8+ T cells, but also in CD4+ T cells, resident memory T cells, and B cells. Moreover, changes in the repertoire at an early stage of treatment seem to be useful for predicting the therapeutic efficacy of ICIs. Further analyses of the repertoire of immune cells are desirable to improve and predict the therapeutic efficacy of ICIs.

Inflammaging and immunosenescence are associated with aging of the human body. Immunosenescence aims to adapt the body systems to aging, while inflammaging is considered as a consequence of immunosenescence. Pająk et al. review the immunosenescence and inflammaging processes in the skin. The triggering factors for inflammaging are UV radiations, changes in the bioavailability of nitric oxide, senescence-associated secretory phenotype factors, or reactive oxygen species, while inhibiting factors such as estrogen, topical rapamycin/metformin, and topical emollients/moisturizers act as geroprotectors and senotherapeutics and can be used as anti-aging treatments. While knowledge on external factors can help people improve their health conditions, knowledge on biochemical factors can help researchers understand the inflammaging process and develop interventions to minimize the impact of aging on the human body [7].

When the skin is overexposed to UV rays, free radicals accumulate in the skin, causing lipid damage and even inducing photoaging of the skin. Yue et al. investigated whether topical treatment with antioxidant drugs of specifically physiological structure may protect skin from photoaging [8]. The encapsulation of shikoninn (C_16_H_16_O_5_, mw 288.31) in β-cyclodextrin (SH-β-CD) delayed the release of the drug and increased drug solubility. SH-β-CD provided the high antioxidant potential and an obvious therapeutic effect on the skin photoaging of mice. The dermal administration of SH-β-CD may be a promising modality in skin disease treatment and skin care.

Chien and Tsai reviewed the pathological effects of mechanical pressure on the skin from the cellular perspective [9]. Cellular responses and interactions, involving fibroblasts, keratinocytes, mast cells, melanocytes, adipocytes, and stem cells, may expedite the propagation and translation of extrinsic mechanical signals of pressure. Under pressure, skin cells and tissue may mechanistically undergo pathological events such as ischemia, chronic inflammation, proliferation, regeneration, degeneration, necrosis, and impaired differentiation. Molecules like yes-associated protein (YAP) are crucial in aiding these processes. Investigations of those mechanotransduction and mechanoresponsive pathways will help determine the pathophysiology of relevant pressure-related dermatoses and tailor cellular- and molecular-oriented management.

Kanekura reviewed the role of CD147/basigin, a transmembrane glycoprotein [10]. CD147 is involved in the development of malignant tumors and T-cell-mediated immunological disorders via the regulation of glycolysis. CD147 binds to monocarboxylate transporters (MCTs) and supports their expression on plasma membranes. Glycolysis is an enzymatic conversion from glucose to pyruvate to produce ATP, and usually occurs under anaerobic conditions, whereas cancer cells depend on glycolysis under aerobic conditions. Human malignant melanoma cells express higher levels of MCT-1 and MCT-4, co-localized with CD147, and homodimerized CD147 associates with two monomers of MCT-1 or 4 and regulates lactate transport. T cells also require glycolysis for differentiation, proliferation, and activation. CD147 is involved in the differentiation from naïve CD4+ T cells into Th17 cells in psoriasis.

Kusumoto et al. investigated photosensitivity in normal skin tissue and hypertrophic scars using normal dermal fibroblasts (NDFs) and hypertrophic scar fibroblasts (HSFs) [11]. *OPN3* encoding opsin 3 is expressed at high levels in both NDFs and HDFs. After the disruption of the peripheral circadian rhythm, blue light (BL) induced peripheral circadian synchronization in NDFs but not in HSFs, although HSFs showed changes in the expression levels of the clock-related genes *PER2* and *BMAL1*. The expression level of *αSMA* encoding α-smooth muscle actin in HSFs was significantly higher compared to NDFs, and was decreased by BL exposure dependently on *OPN3*. BL might be applied for the prevention and treatment of hypertrophic scars and keloids.

The articles in this Special Issue provide novel insights into the ongoing research of skin diseases. We earnestly hope these articles will help advances in the prevention, diagnosis, and treatment of a variety of skin diseases.

## Data Availability

No new data were created or analyzed in this study. Data sharing is not applicable to this article.
